# Fast antibody fragment motion: flexible linkers act as entropic spring

**DOI:** 10.1038/srep22148

**Published:** 2016-03-29

**Authors:** Laura R. Stingaciu, Oxana Ivanova, Michael Ohl, Ralf Biehl, Dieter Richter

**Affiliations:** 1 Jülich Centre for Neutron Science JCNS, Forschungszentrum Jülich GmbH, outstation at SNS, Oak Ridge National Laboratory, Oak Ridge, TN 37831, USA; 2 Jülich Centre for Neutron Science JCNS, Forschungszentrum Jülich GmbH, outstation at MLZ, 85747 Garching, Germany; 3 Jülich Centre for Neutron Science JCNS (JCNS-1) & Institute of Complex Systems (ICS-1), Forschungszentrum Jülich GmbH, 52425 Jülich, Germany

## Abstract

A flexible linker region between three fragments allows antibodies to adjust their binding sites to an antigen or receptor. Using Neutron Spin Echo Spectroscopy we observed fragment motion on a timescale of 7 ns with motional amplitudes of about 1 nm relative to each other. The mechanistic complexity of the linker region can be described by a spring model with Brownian motion of the fragments in a harmonic potential. Displacements, timescale, friction and force constant of the underlying dynamics are accessed. The force constant exhibits a similar strength to an entropic spring, with friction of the fragment matching the unbound state. The observed fast motions are fluctuations in pre-existing equilibrium configurations. The Brownian motion of domains in a harmonic potential is the appropriate model to examine functional hinge motions dependent on the structural topology and highlights the role of internal forces and friction to function.

Antibodies are large Y-shaped glycoproteins produced by the humoral immune system and are generically called immunoglobulins (Ig). They consist of three equal-sized fragments (see IgG1 in Fig. 1) connected by a flexible linker region: two antigen-binding fragments (Fab) and one constant fragment (Fc). Each Fab fragment has a constant region (C) attached to the linker region and a variable antigen-binding region (V) that accounts for the specificity of an Ig molecule to a target^1^. The Fc fragment imparts signalling and effector functions. The flexibility and fragment motion seem to be crucial to understand the functionality of antibodies. Therefore, over the years, an extensive amount of research has been performed in order to understand the structure and flexibility^2^,^3^,^4^,^5^,^6^,^7^. Specificity and constancy make antibodies attractive for use in immunotherapy. They have been used to develop new drugs targeting specific cells for inhibition/activation of cell processes, as antibody-dependent cellular cytotoxicity or phagocytosis^1^, and to deploy specific drugs by immunoliposomes^8^ or radiation therapy^9^.

Antibody fragments are built from four peptide chains joined together by disulphide bonds. Two heavy chains (M_w _~ 50 kDa), joined by disulphide bonds, form the Fc fragment from about half of their length. Two shorter light chains (M_w _~_ _25 kDa) complement the other half of the heavy chains to build up the Fab fragments. The linker region is responsible for the high flexibility between the 3 fragments and allows Fab to bind to antigens of various shapes while the Fc fragment simultaneously can bind to a receptor or complement. The linker region has three components^10^. The core segment contains a CPPC amino acid motif connecting the heavy chains with several disulfide bonds between the cysteines (C) and proline pairs (PP) that make this motif rigid (IgG4 has sequence CPSC with serine (S)). The flexible upper and lower linker regions connect the Fab and the Fc fragments to the core, respectively. While the upper linker regions influence the Fab-Fab flexibility, the lower linker regions influence the Fab-Fc flexibility.

Variations in the Fc fragment distinguish the five major classes of immunoglobulins. Among those, IgG is the most abundant in serum, with four subclasses numbered according to their abundance. The subclasses differ in the length of the linker region and how many disulfide bonds link the chains^11^. IgG1, IgG2 and IgG4 with about 60%, 32% and 4% abundance, respectively, have a similar short linker region with two or four disulfide bonds. IgG3, with about 4% abundance, has a longer linker region with eleven disulfide bonds. IgG preparations from serum may contain monomeric and dimeric populations in dynamic equilibrium, where dimers may also have higher activity, e.g. for intracellular antigens^12^. IgG may be considered as a good general model for studying immunoglobulins, as it is also a model for the majority of immune drug developments.

Small-angle X-ray and neutron scattering (SAXS/SANS) are used to examine the global conformation of different species of antibodies in solution. Conformations depend on species, type and buffer solvent observed, with a high degree of variability^2^,^3^,^4^,^13^.

The dynamics of IgG antibodies are difficult to explore since most experimental methods are limited to conformationally averaged structures, like SAXS/SANS or with artificially frozen configurations for electron microscopy or crystallography. Fluorescence anisotropy can be used to examine the rotational diffusion of an attached chromophore. Ref.^14^ showed correlation times of 168 ns attributed to the global motion of the entire rabbit IgG molecule together with shorter correlation times of about 33 ns attributed to faster motion of Fab arms over an restricted angle. This reinforced the model of an IgG molecule with flexible joints at the junction of the Fab segments^15^. Ref.^16^ and^17^ found similar rotational correlation times of hundreds of nanoseconds that were attributed to global motions of IgG molecules. Several years later, similar antibodies from rabbit were re-examined using fluorescence spectroscopy and the new data indicated that the observed long correlation times were not from tumbling of the whole IgG molecule but rather from large domain motions, e.g. the Fab arms wobbling over the hinge region^18^. This led to a new dynamic model of the IgG in which large protein domains like Fab arms are not restricted to movements over small angles but are very flexible.

Given such a large molecule, it is a challenge to determine what types of intramolecular motions can be observed and to relate these motions to overall functionality. Neutron Spin Echo (NSE) is a high-resolution space-time resolving spectroscopy that reveals information on time scales from about 10 ps to 500 ns and covers length scales from fractions of a nm to the size of the molecule and beyond, with the advantage that native proteins without special markers are used^19^,^20^. NSE has already been successfully applied to investigate the internal dynamics of phosphoglycerate kinase, alcohol dehydrogenase^21^,^22^ and intrinsically disordered proteins^23^. A first NSE study on IgG^24^ measured the effective diffusion coefficient of pig IgG in solution and showed that some domain motions were present. From a structural perspective, new research involving SAXS shows a strong conformational diversity of IgG molecules in solution with both compact and more “open” conformations and reports angular ranges between the Fab-domains of 100–166°^25^. A recent combined fluorescence anisotropy and molecular dynamics simulation study finds highly flexible IgG molecules. Simulations suggest strongly correlated inter-domain motions such that antibody molecules can form different contacts for different inter-domain motions^26^.

We report a detailed study of human IgG domain motions using NSE spectroscopy on a protein solution in order to resolve the motional pattern in space and time. At long Fourier times (t > 15 ns) we observe translational and rotational diffusion with a clear signature of internal dynamics at shorter times. The internal dynamics are related to the fragment motions held together by the linker region and show characteristics of Brownian motion in a harmonic potential. We access information about fragment relaxation time, the force constant related to the flexible linker region, motional amplitudes and the friction exerted on the fragments. NSE is a unique technique that is able to deliver this detailed information, which cannot be accessed by other methods. The results may help to develop new or improved strategies for drug design with the ability to include specific details on the domain dynamics within this complex and unique protein class.

## Results

### Conformation of IgG in solutions

We used X-ray scattering to investigate the configurations of IgG in solution in a concentration range between 3 and 26.4 mg/ml. Lyophilized IgG powder from human serum was reconstituted in a D_2_O buffer, thus containing all subclasses of IgG. The measured background corrected and concentration (*N/V*) normalized intensities are shown in Fig. 2a. The scattering intensity *I*(*Q*) of *N* identical particles is proportional to *I*(*Q*) ~ *NP*(*Q*)*S*(*Q*,*c*) with the orientation averaged, concentration-independent form factor *P*(*Q*) containing information about the shape of the protein and the concentration-dependent structure factor *S*(*Q*,*c*) containing information about the protein-protein interaction as


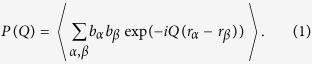



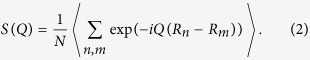


Here b_α_ are the scattering amplitude of atom *α* at position *r*_α_ and centre of mass positions R_n_ within the ensemble average indicated by <> and *Q* represents the solid scattering angle. The scattering length b_α_ is the coherent scattering length for neutron scattering or a *Q* dependent scattering amplitude^27^ with *b*_α_(0) *=* *Zr*_e_ for SAXS, where *r*_e_ is the classical radius of the electron and *Z* is the electron number of the atom. The form factor of a protein can be calculated from the atomic coordinates, if a structure is known e.g. from the PDB databank^28^. For hard sphere interactions, the structure factor can be calculated in terms of the Percus-Yevick approach^29^,^30^. For additional screened coulomb repulsion a MSA structure factor^31^,^32^ is expected as observed for many compact proteins in buffers with high salinity^33^,^34^. An attractive component is observed if trivalent salts are added to the buffer^35^. For non-compact and asymmetric particles an apparent structure factor *S*´(*Q*) has to be used dependent on the scattering amplitude 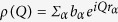
and the asymmetry factor *β*(*Q*)^36^


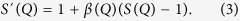


with


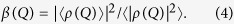


**Figure 1:**
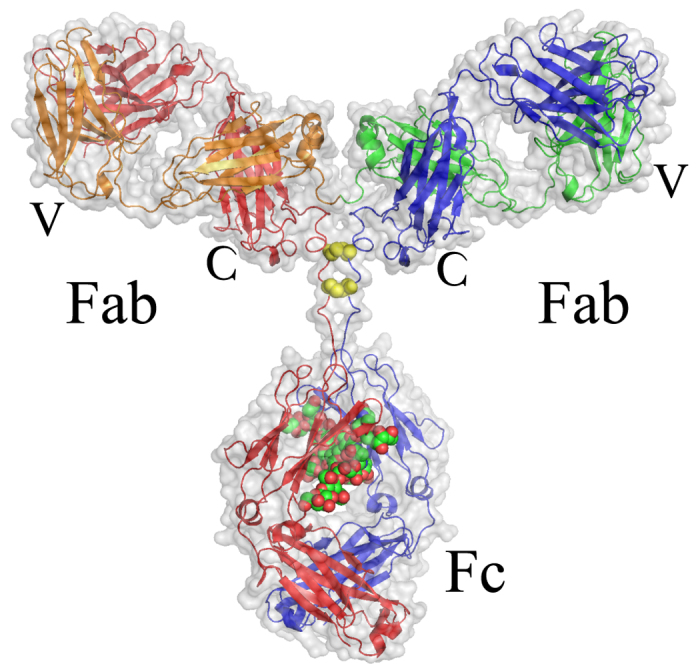
Immunoglobulin G1 with Fc and two Fab fragments with the van der Waals surface in grey. The Fc fragment is build from parts of the heavy chains (red and blue cartoon) and the glycans (red and green spheres) between the two heavy chains. Fab fragments are built from heavy and light chains (orange and green cartoon) with a hinge between the variable region (V) and the constant region (C) of the Fab fragment. The heavy chains are connected in the linker region by two disulfide bonds (yellow spheres). The structure displayed is based on the human IgG1 structure IgG-ALL.pdb of Padlan^37^. IgG-ALL is a composite model with the Fc fragment from 1FC2.pdb and the Fab fragment of 2IG2.pdb^49^,^60^. Details, as the flexible linker region, are modeled to display a complete structure including the linker region. Figures created with PyMOL (www.pymol.org).

From a measurement of several concentrations the form factor and structure factor can be separated (see Methods). In order to obtain a description of the form factor in terms of an atomic model we modified the human IgG monomer structure taken from IgG-ALL.pdb^37^ (see Fig. 1) to account for the major subclasses IgG1 and IgG2. We allowed for bending and stretching of the linker region with rigid Fab and Fc fragments (see Methods for details). It was not possible to model the scattering data by the monomer alone. However, the dynamic light scattering (DLS) data showed a narrow peak (about 30% width) so only an additional dimer could be present in our sample, which cannot be discriminated within DLS. Modelling with an additional Beaucage function results in a second species with a radius of gyration *R*_g_ = 7.4 nm. This is slightly larger than the IgG-ALL.pdb *R*_g_ of 5.7 nm but significantly smaller than a dimer with a connection at the variable region of the Fc fragment with *R*_g_ of 10.4 nm. Ref.^38^ proposes head-to-head double Fab arm bound dimers or single arm bound dimers. Using the monomer as a template, we modelled the double arm bound dimer with interlocked Fab fragments with arbitrary touching points resulting in *R*_g_ = 7.4 nm as a prototype. Accordingly, the measured form factor is a weighted sum of the form factors of dimer and monomer, and the structure factor is an apparent structure factor including contributions between monomers only, dimers only and between monomers and dimers. In a least squares fit procedure of the mixed form factor, we allow the Fc fragments of the dimer to move around the linker regions. For the monomer, we allow bending and stretching of all fragments around the linker region. Fig. 3 shows the result of the fitting procedure with the individual contributions of monomer, dimer and a residual background together with the corresponding resulting structures. We find a 48% fraction of monomers incorporated into dimers. Dimers show a small bending of the Fc fragments, resulting in *R*_g_ = 6.4 nm. The monomer mainly presents a bending of the Fc fragment to one of the arms, resulting in a more compact structure with *R*_g_ = 4.8 nm compared to the starting structure. This value is very close to 4.9 nm for IgG1 and 4.7 nm for IgG2 of ref.^39^. Although we typically obtain a single conformation describing the SAXS data reasonably well, this conformation has to be considered as a representative for the ensemble present in solution. Ensemble fitting, as in the work of ref.^39^, can result in similar quality fits but may be biased by the choice of flexible connections or by the fact that we also observe dimers. The static picture of a small angle scattering measurement interpreted by a single conformation or a minimal ensemble cannot show directly any dynamics or conformational change. In our single conformation analysis the fit errors of individual bending angles (see Materials and Methods) are large for the monomer *α*_*2*36_ = 26° ± 23°, *α*_241_ = −44° ± 23°, *α*_247_ = −50° ± 28° showing a high degree of flexibility within an acceptable quality of the fit. We might interpret this as a sign of flexibility within the conformation. Nevertheless, the monomer and dimer structures can be used as models to determine the diffusion constants of IgGs.

**Figure 2:**
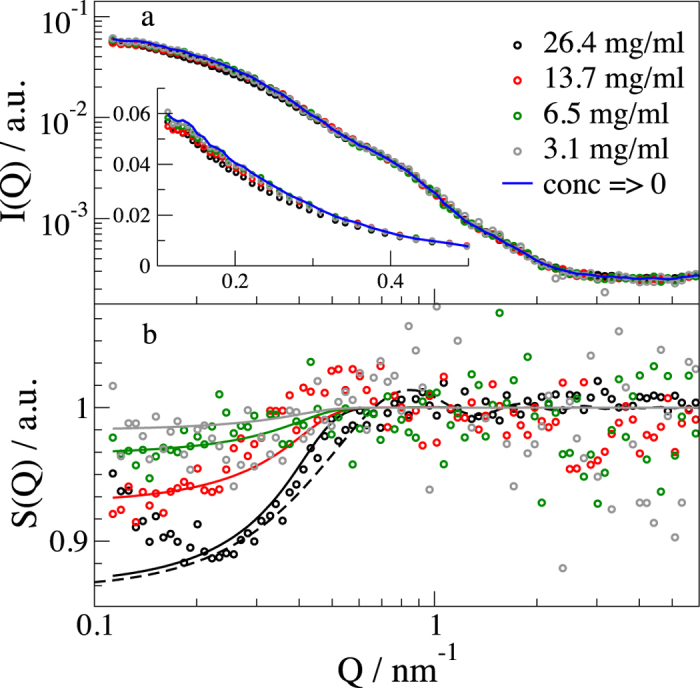
IgG SAXS scattering profiles. **(a)** Background corrected and concentration scaled SAXS scattering profiles of IgG. The solid line represents the zero concentration as the form factor. The inset shows the low Q data in linear scale to increase visibility of the structure factor effect. **(b)** The structure factor extracted by dividing with the form factor. Solid lines represent a fit corresponding to the MSA structure factor including the asymmetry. The dashed black line shows the effect of the asymmetry factor β. For the highest concentration, a small effect of aggregates leads to an increase in the structure factor, which is not observed for lower concentration.

Fig. 2b shows the resulting structure factor for all concentrations. The structure factor is Percus-Yevick like with a hard sphere radius of 3.5 nm, not too far from the results of Scherer *et al.* with 4.4 nm by light scattering^40^. As seen for the highest concentration, the asymmetry of IgG described by the asymmetry factor *β*(*Q*) has a small influence at low *Q* but already suppresses the first structure factor peak around 0.9 nm^−1^ and the following peaks completely. At high *Q* we observe a plateau in the form factor as residual background, which is about two orders of magnitude below the measured buffer background.

### NSE results

Figure 4 displays the intermediate scattering function *I*(*Q,t*)/*I*(*Q*,*t* = 0) as measured by NSE. Already by simple inspection, a clear deviation from a simple diffusion process is observed on short times. Fitting the data by the corresponding single exponential decay *I*(*Q*,*t*)/*I*(*Q*,*t* = 0) = exp(−*D*_eff_ *Q*^2^*t*) one can see a proper fit for long times *t* with an effective diffusion coefficient *D*_eff_. Also, on a ten nanosecond scale, clear indications for a faster relaxation can be observed, which we attribute to motions within the molecule.

### Separation of global and internal dynamics

The coherent intermediate scattering intensity *I*(*Q*,*t*)/*I*(*Q*,0) measured by NSE on a protein in solution has contributions from overall translational and rotational diffusion and from motions within the molecule that we call internal dynamics. In the general case, all motions might be coupled. In a simplifying assumption, known as a decoupling approximation, we assume that the internal dynamics (int) do not alter the overall diffusion and that translational (trans) and rotational (rot) diffusion are decoupled too. For the single particle pair correlation function or coherent scattering function we assume


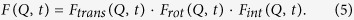


### Global diffusion

The diffusional terms for a single rigid protein can be described by


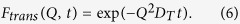



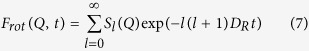


with


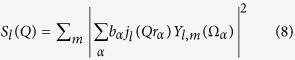


where *S*_l_(*Q*) are the coefficients of a multipole expansion of the asymmetric form factor with scattering length *b*_α_ of the atom *α* at position *r*_α_ and orientation *Ω*_α_, *j*_l_(*Qr*) are the spherical Bessel functions and *Y*_l,m_(*Ω*_α_) the spherical harmonics^20^,^41^. The internal dynamics will be discussed later. The measured NSE signal is independent of direct concentration effects as *I*(*Q*,*t*)/*I*(*Q*,0) is normalized by *I*(*Q*,*t* = 0). For larger concentrations the protein scalar translational diffusion coefficient *D*_*T*_ is influenced by direct interactions subsumed in the structure factor *S*(*Q*) and hydrodynamic interaction described by the hydrodynamic function *H*_T_(*Q*). This leads to the correction *D*_*T*_(*Q*) = *D*_*T0*_*H*_*T*_(*Q,c*)/*S*(*Q,c*)^42^,^43^. The hydrodynamic function *H*_*T*_(*Q*) is found to be constant for flexible proteins^20^,^21^,^22^ and reduces the translational diffusion coefficient with *H*_T_(*Q*) = *H*_T_(*Q* *=* *∞*) = *η*_0_/*η *< 1^44^,^45^,^46^. *η*_0_/*η* is measured as the ratio between solvent viscosity *η*_0_ and protein solution viscosity at the measured concentration *η*. Hydrodynamic effects also influence the rotational diffusion coefficient subsumed in the hydrodynamic factor H_R_ for rotational diffusion. According to ref.^47^ for spherical particles, this effect can be estimated as 1*−H*_R_ = (1−*H*_T_(*Q* = ∞))/3. For the strongly asymmetric IgG this may be different. From the 6 × 6 diffusion tensor ***D*** the effective single rigid protein diffusion coefficient *D*_0_(*Q*) comprising rotational and translational diffusion together can be calculated by^19^


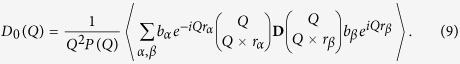


**Figure 3:**
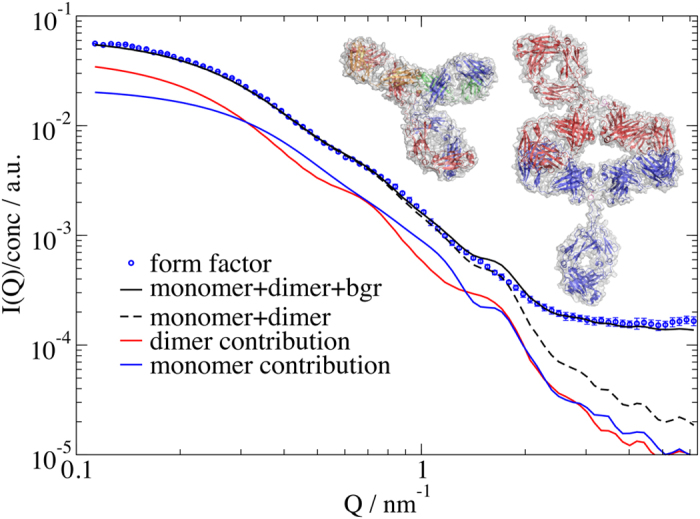
The SAXS form factor with the fit result. The form factor fit includes 52 ± 4% monomer as monomer and 48 ± 4% monomer in dimers and a background contribution (bgr). The dashed line shows the combined monomer-dimer scattering, the red line shows the contribution of the dimer, while the blue line shows the monomer, all without the residual background. The remaining peak at 1.65 nm^−1^ might be due to bending inside of the Fab fragments. The graphics show the resulting structure of the monomer with colouring of the chains as in Fig. 1 while the two chains of the dimer are coloured red and blue, both with a grey surface. Figures created with PyMOL (www.pymol.org).

The 6 × 6 diffusion tensor ***D*** was calculated by HYDROPRO on basis of a valid PDB conformation^48^. As result we get the scalar diffusion coefficients *D*_T0_ = 3.48 × 10^−2 ^nm^49^/ns and *D*_R0_ = 6.96 × 10^−4 ^ns^−1^ for the monomer and *D*_T0_ = 2.71 × 10^−2 ^nm^49^/ns and *D*_R0_ = 3.53 × 10^−4 ^ns^−1^ for the dimer. The rotational correlation times *τ*_*r*_=1/6*D*_*R*0_ for the monomer and dimer are on a scale of 260 ns^−1^ respectively 500 ns^−1^ and are longer than the translational diffusion times (e.g. 29 ns at Q = 1 nm^−1^). Therefore, we can extract the effective diffusion *D*_eff_(*Q*) comprising translational and rotational contributions of the overall protein by applying a single exponential fit for times t > 15 ns. The result is shown in Fig. 5 together with the DLS result for the same concentration as used for NSE measurement. We use equation (9) and the 6 × 6 diffusion tensor ***D*** together with the monomer and dimer structure to calculate *D*_0_(*Q*) of the rigid structures (see inset in Fig. 5). The increase from low *Q* to the higher *Q* values results from the stronger visibility of the rotational diffusion, if the observation length scale (2*π/Q*) reaches the size of the protein. At low *Q* we see only the translational diffusion as measured by DLS. The expected diffusion *D*_eff_(*Q*) of the monomer/dimer mixture can be calculated as the average of monomer and dimer diffusion weighted by the scattering contribution to the form factor as shown in Fig. 3. To include the hydrodynamic effects, the translational contribution *D*_eff_(*Q* *=* 0) is corrected by *H*_T_(*Q* *=* *∞*) = 0.66 as calculated from viscosity and correspondingly the rotational diffusion contribution *D*_eff_(*Q*)*–D*_eff_(*Q* *=* 0) as described above by *H*_R_. At higher *Q* we observe a coincidence with the experimental data including only the hydrodynamic correction (see Fig. 5). The additional correction by the structure factor (1*/S*(*Q*)) leads to an upturn at low *Q* and results in an excellent agreement with the DLS measured diffusion coefficient. We note that the excellent agreement of the model and the diffusion coefficients determined from NSE is achieved without fitting of any parameter and proves that the long-time dynamics is attributed to an overall translational and rotational diffusion.

### Internal dynamics

The specific structure of IgG with three nearly equal sized fragments connected by flexible linkers suggests a simple model of three fragments within a harmonic potential. The flexible linkers may act as springs fixing the relative equilibrium position of the fragments but allowing fluctuations around the equilibrium position. The model is not restricted to IgG but may be used for any protein structure that can be described by relatively rigid domains connected by flexible linkers taking into account the specific three-dimensional arrangement of the fragments and can include additional internal motions in the connected domains (for details see Methods).

We consider Brownian motion of the fragments in a harmonic potential around the equilibrium position 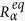, a problem described by the Ornstein-Uhlenbeck process^50^,^51^. The coherent intermediate scattering function 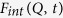 is (for details see Methods)


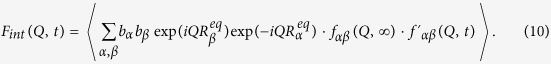


Here f_αβ_(Q,∞) is a time independent Debye–Waller like factor. The time dependent part is


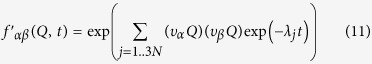


with displacements 
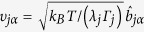
 in Brownian normal mode *j* with relaxation time 1/*λ*_*j*_ and friction *Γ*_*j*._ The contribution of internal dynamics to the NSE signal is 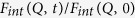. The dynamics of single independent particles or subunits can be described by the much simpler function (see Methods)


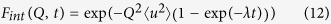


with the mean square displacement <u^2^> in the harmonic potential^52^. Nevertheless, in this simplification, the specific arrangement of the conformation is lost.

**Figure 4:**
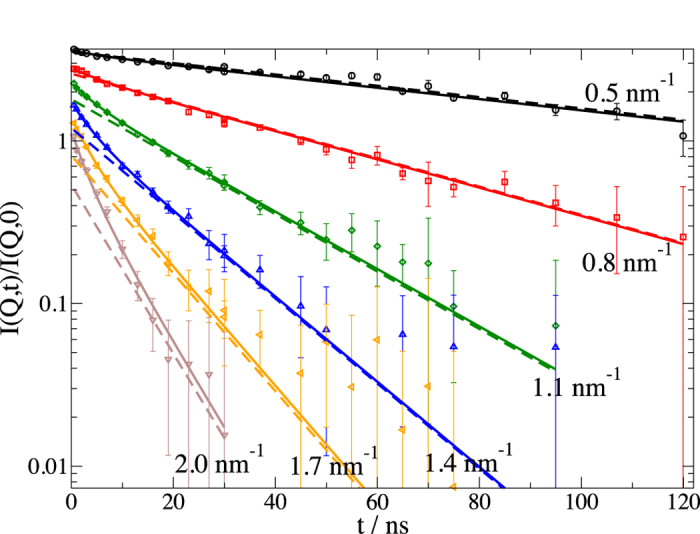
Intermediate scattering functions *I*(*Q*,*t*)/*I*(*Q* **,0) of IgG.** The *Q* values are 0.5 nm^−1^, 0.8 nm^−1^, 1.1 nm^−1^, 1.4 nm^−1^, 1.7 nm^−1^and 2.0 nm^−1^ as indicated. Spectra were shifted for clarity upwards by a consecutive factor of 1.3, with the highest Q not shifted. Solid lines in the same color represent the fit by equation (5) with contributions from translational and rotational diffusion from equation (6) & (7) and internal dynamics described by equation 10 with 9 degrees of freedom as described in the text. Dashed lines correspond to the contribution of translational and rotational diffusion and are close to a single exponential for times t > 15 ns. The amplitude of internal dynamics is the difference between the contribution of diffusion and the observed signal, respectively the solid line of the fit curve.

**Figure 5:**
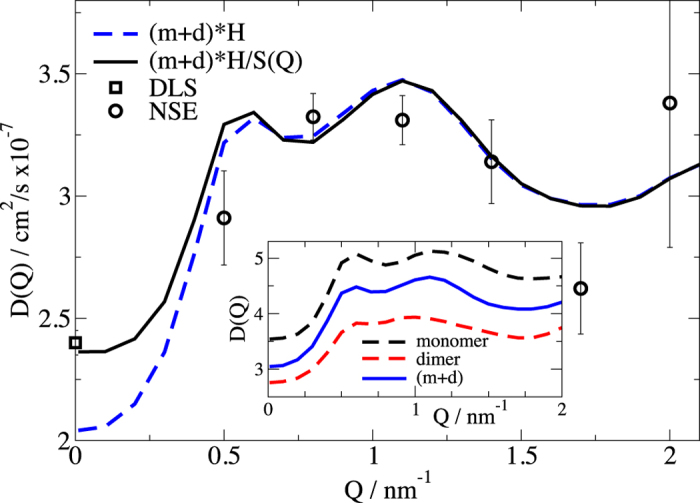
Effective diffusion coefficient *D*_eff_ **for long Fourier times.** *D*_eff_ (black circles) is the result of fitting *I*(*Q*,*t*)/*I*(*Q*,*0*) = exp(*−Q*^2^*D*_eff_*t*) for t > 15 ns to the concentration of 29 mg/ml. Black square shows the effective diffusion for low *Q* as measured by dynamic light scattering at the same concentration. The dashed blue line shows the result for *D*_eff_(*Q*) as a mixture between monomer and dimer including the effect of hydrodynamic interactions. The black solid line additionally includes the structure factor correction. The inset shows the diffusion coefficients for monomer and dimer with the intensity averaged *D*_eff_(*Q*).

Instead of normal modes that are based on an elastic network model^53^ with a simplified force field, we chose a reduced set of obvious displacements patterns. In the first model, we consider three perpendicular vectors of unit length for each of the three fragments. One is aligned parallel to the linker from the IgG centre of mass to a fragment centre of mass, the second is aligned perpendicular to the plane spanned by the fragments centre of masses and the third is perpendicular to both, laying inside the plane. The nine displacement vectors were used as vibrational and Brownian modes with equal amplitude and represent nine degrees of freedom (*dof* 9). The diagonal elements of the friction matrix are equal for all subunits and we use one friction parameter for all. In a second model, we consider a less flexible linker region assuming a length restriction of the linker with forbidden movements along the linker (*dof* 6). The simplest model, according to equation (12), assumes independent movements of the fragments but results only in the mean square displacement (*msd*). Fig. 4 shows NSE data and the fit according to equation (5) with contributions from translational and rotational diffusion from equation (6) & (7) and internal dynamics described by equation (10). For all models the translational diffusion *D*_T_ is fixed to the corrected HYDROPRO result. *H*_R_ can only be estimated for spherical particles. Here we allow it to be fitted together with the relaxation time and the friction coefficient determining the displacements 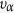. We find an excellent description of the data independent of the model used, with parameters shown in Table 1.

*H*_R_ is smaller than calculated for hard spheres with 1−(1–0.66)/3 = 0.88, but in reasonable agreement considering the strong asymmetry of IgG reflecting a stronger hydrodynamic interaction. The relaxation time 1*/λ* is, within the error bars, equal. The displacements 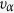 for the models *dof* 9 and *dof* 6 have a comparable root mean square displacement (*rmsd*) as found in the simpler *msd* model.

The friction of the *dof* 6 model is reduced compared to the *dof* 9 model to allow larger amplitudes and compensate the smaller number of degrees of freedom with about equal *rmsd*. The friction of a fragment is the sum over all atoms and differs between Fc and Fab fragments due to the different size. The friction of a non-linked fragment in solvent can be determined by *γ* = *k*_*B*_*T*/*D*_*T*_ with the Boltzmann constant *k*_B_ for diffusion at temperature *T*. D_T_ of the single fragments calculated by HYDROPRO are on the order of 0.06 nm^2^/ns, leading to a friction of 42500 g/ps/mol and 39200 g/ps/mol for the unbound Fc and Fab fragments, which are very close to the total friction of the bound fragments within the *dof 9* model.

We can calculate a force constant for the linker as *k*_*j*_ = *λ*_*j*_*Γ*_*j*_ for the *dof* models and may estimate a corresponding force constant from equipartition for the *msd* model as *k* *=* *k*_B_*T/rmsd*^2^. We can compare the values given in Table 1 to the force constant of an entropic spring *k*_e_ *=* 3*k*_B_*T/N/b*^2^. With a monomer length *b* of 0.38 nm per amino acid corresponding to the average distance between C_alpha_ atoms and a length *N* *=* 8 residues from the disulfide bonds to the fragment, the entropic force constant is 6.4 g/ps^2^/mol. Eight segments seem too short to build an entropic spring but even accounting for a twice larger effective length (*N* = 16) does not change the entropic spring force constant significantly. From the above we can summarize that the linker region acts like a spring. Considering that the friction for the *dof 9* model is close to the unbound fragment friction and the force constant is close to an entropic spring, the *dof 9* model needs to be favoured.

Estimating the influence of the dimers present, we may assume an extreme case: Within the *msd* model the average mean square displacement is proportional to the number of fragments contributing to the amplitude. As the dimer is still active^38^, we can assume that the fragments are partially mobile with the only restriction that the adhered fragments are less mobile or stiff and cannot contribute to the mean square displacement. In this case, 4 bound fragments in a dimer are stiff and 2 are movable. Consequently, 68% of the fragments are mobile and 32% are not flexible and cannot contribute to the observed dynamics. This can be compensated by a 21% increase of the displacement amplitude in the *rmsd*.

On the other hand, we may compare the expected force at 1 nm displacement (10.7 pN) to the unfolding force of some proteins’ secondary structure. The myomesin domain My6 unfolds at about 200 pN, titin unfolds between 150–300 pN, while spectrin unfolds at about 30 pN^54^. The observed force is able to unfold loosely bound secondary structure but seems to stay below the ability to unfold even weaker bound secondary structure.

### Amplitude of internal motions

**Figure 6:**
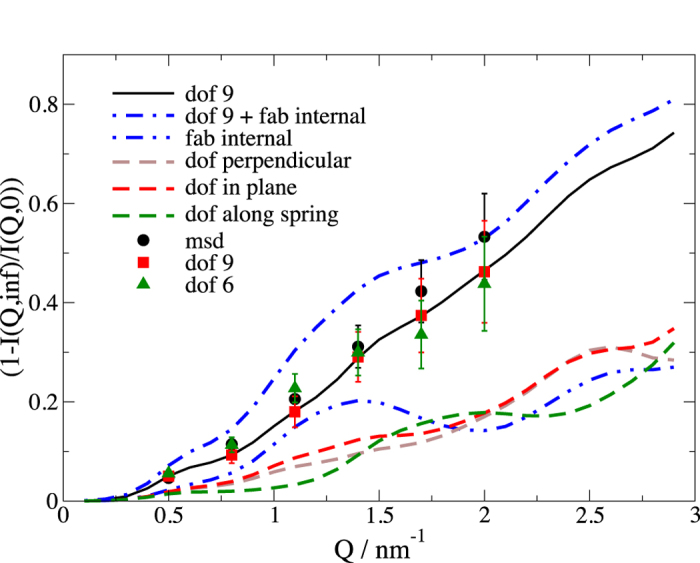
Amplitude of internal motion as 1*−F*_int_(*Q,*∞)/(*F*_int_(*Q*,0). The result of various models describing the internal dynamics by nine degrees of freedom (*dof* 9, red squares), as independent particles (*msd*, black circles) and by six degrees of freedom with the displacements along the spring fixed (*dof* 6, green triangle). The dispersion of 9 (black solid line) is composed of the individual contributions of displacements perpendicular (dashed, grey) and in the plane (dashed, red) and along the linker (dashed, green). Restricting the movements along the linker to six *dof* shows smaller amplitudes at larger *Q* corresponding to the stronger contribution of the movements along the linker above 1.3 nm^−1^. Adding hinge bending within the Fab fragments to the *dof* 9 model (dash-double-dotted, blue) shows stronger amplitude around 1.3 nm^−1^ in correspondence to the single contribution (dash-dotted, blue). A fit with this model reduces the hinge contribution to small amplitudes.

The amplitude of the internal dynamics within the NSE signal can be evaluated from the ratio of the internal contribution *F*_int_(*Q,t*) at infinite time and *t* = 0. Amplitudes are shown in Fig. 6. In general, we observe an increasing amplitude that follows basically an exp(*Q*^2^<*u*^2^>) dispersion as expected according to the simple model of a free particle of equation (12). Using *dof* 9 we find similar, but slightly smaller, amplitude due to interference between the fragments. Restricting to *dof* 6, we find a smaller contribution at larger *Q* with a larger contribution at lower *Q*. Inspecting the individual contributions with respect to the displacement direction, we observe that the displacement along the linker has a stronger contribution at *Q *> 1.3 nm^−1^, which is missing for larger *Q* in the restricted model. Accordingly, the fitting procedure increases the amplitudes to result in an overall good fit.

To test whether we can observe an additional contribution from hinge bending in the Fab fragment we allow for bending of the variable region (V) against the constant region (C) of the Fab fragments. With this movement included into the model, the fit leads to negligible amplitude for this bending intra Fab displacement. In order to show how such a displacement would reveal itself in the experimental results, we assume a bending contribution of ±30°. In Fig. 6 this additional contribution gives rise to peaks contributing between 1 and 1.5 nm^−1^, which is not seen in the NSE measurements. We therefore conclude that such bending amplitudes are quite small, or possible conformational changes are much slower than the observed timescale.

The angle between fragments can be defined as the angle between the fragment centers of mass with the disulfide bonds in the linker as the origin, which are in fact close to the center of mass of the IgG-ALL.pdb. For the IgG-ALL.pdb structure the angle between the Fab fragments is 103° and from Fab to Fc fragments it is 128°. The angular amplitude for the *dof* 9 model is about 5° for each fragment and around 6° for the restricted *dof* 6 model. The “in plane” angle provides the main contribution to the angular displacement between the fragments. Symmetric and asymmetric deformations lead to an angular range ±10° between two fragments. Consequently, the full width in angular range is about 20° for displacements within an average energy of k_B_T in the harmonic potential. If we allow 3 k_B_T to include 3^1/2^ times the width of the Gaussian distribution in the harmonic potential (about 95% of the allowed deformations), then we obtain about 35°. Ref. 25 reports within an ensemble optimized fitting (EOM) analysis of SAXS experiments an angular range from 100°–166° between the Fab fragments and 56°–124° between Fab-Fc fragments. Ref.^55^ reports 115°–172° between the Fab and 66°–123° to the Fc fragment from crystallographic analysis. Ref.^39^ classifies less accurately between more T-shaped and Y-shaped conformations. Both of the first analyses try to define sets, which reflect a minimal to maximal allowed range that are about 2–3 times larger compared to our results. Ref.^25^ finds a bimodal distribution of sizes interpreted as “open” or “closed” configuration in structural equilibrium^25^. These “open” or “closed” structures are interpreted within the pre-existing equilibrium model initially formulated by Pauling. Different coexisting configurations are assumed to exist in equilibrium exhibiting different binding sites and binding specifity^56^. The pre-existing equilibrium model was shown to be valid for antibodies in ref.^57^ for different IgG1 monoclonal antibodies and in ref.^58^ for SPE7. EOM seems to capture an average picture of all equilibrium configurations and might give a picture of different pre-existing configurations. However, in contrast to the EOM method, our measurement is a direct determination of displacements and force constant in an equilibrium configuration that is also independent of the actual mean configuration. The relaxation time of about 6.3 ns seems quite fast but is comparable to the necessary time for the free fragment to diffuse over a length of the *rmsd* we found.

## Conclusion

This study focused on the fragment motion of IgG. In the experiment, aside from strong diffusion contributions, a clear signature of the internal dynamics on a timescale of 6–7 ns was observed. The combination of translational and rotational diffusion of the rigid protein describes the observed long time dynamics on an absolute scale, despite the fact that we have a mixed population of monomers and dimers, as found in serum.

The fragment motion shows itself in terms of a strong decay of *S*(*Q,t*) at short times, well separated from the overall diffusional relaxation. The data were analysed in terms of an Ornstein-Uhlenbeck like relaxation within a harmonic potential. Such an approach neglects the details of the complex linker region interaction on the residue level. Nevertheless, the dynamics is described in an excellent way. Even for a short flexible linker the ensemble average seems to be sufficient to produce the characteristics of a harmonic spring. The obtained spring constant appears to be realistic compared to an entropic spring of similar length. The resulting forces stay below the limits that would be necessary to unfold the secondary structure of the attached fragments. The observed effective friction appears to be close to the friction of a free unbound fragment. None of the directions seems to be suppressed.

The pre-existing equilibrium hypothesis^56^, with multiple local minima in the configurational energy landscape, will have long transition times between the minima but is compatible with fast 6–7 ns motions in the local minima. Consequently, NSE observes the fast dynamics in pre-existing equilibrium states. The conformational flexibility in a pre-existing equilibrium configuration might be needed to adapt faster to a specific antigen when the antigen approaches the binding site of a fragment.

NSE delivers unique, direct information about the internal dynamics of IgG, which cannot be resolved by any other technique. The Ornstein-Uhlenbeck model is able to give information about amplitude, friction, and force constant of domains bound by flexible linkers or for domain motions with more complex joint topology. Therefore, different hinge types dependent on the protein structure can be directly compared within the same model. For IgG the key questions for the future will be how the buffer environment or mutations to IgG influence the dynamics. Also, the dynamics of the various types of immunoglobulins can be examined. This information may be relevant to understand how monoclonal antibodies can be used as drugs and how to improve biocompatibility and bioavailability.

## Methods

### Sample preparation and characterization

IgG from human serum was purchased from Sigma-Aldrich as lyophilized powder (Prod. Code 56834) and reconstituted in a 50 mM sodium phosphate D_2_O buffer with 0.1 M sodium chloride at pH 6.6. All measurements were done at 25 °C. Samples were centrifuged overnight to remove any larger aggregates. Samples with 3.1, 6.5, 13.7 and 26.4 mg/ml concentration were prepared for SAXS measurements. For the NSE experiment, one sample was prepared with a concentration of 29 mg/ml. Protein concentration was determined by absorbance at 280 nm with E^1%^ = 14. Protein solutions for NSE were dialyzed for 24 h of equilibration within the D_2_O buffer. As a background sample, we used the dialysate after equilibration to account for exchanged hydrogen atoms. Dynamic Light Scattering (DLS) measurements (Zetasizer Nano, Malvern) on a concentration series from 0.8 to 29 mg/ml were performed to observe the size distribution, detect possible aggregation and to obtain the diffusion coefficient of IgG for infinite dilution by extrapolation to zero concentration (see supplementary information). Larger aggregates were only observed in negligible amount during DLS measurements. The width of the observed peak was about 30% typical for monomeric solutions. The experimental DLS diffusion coefficients as a function of protein concentration are shown in supplementary Fig. S1a. Viscosity measurements (rolling ball viscometer, Lovis 2000, Anton Paar) also were performed for the same concentration series to determine the intrinsic viscosity. Viscosity experimental data are shown in supplementary Fig. S1b.

### Small-Angle X-ray scattering

SAXS was measured on a Kratky type SAXS instrument (SAXSpace, Anton Paar) at FZ-Jülich with X-ray wavelength of 0.154 nm. Samples were loaded in quartz capillaries of 1 mm diameter and measured for 1 h counting time per sample. The measured intensities were corrected for dark current, empty cell and solvent scattering following standard procedures and desmeared according to the built-in Lake algorithm within the SAXQuant software (Anton Paar). Data were averaged over *Q* to achieve a smaller noise at larger *Q*.

Form factor and structure factor are separated by a self-consistent optimization procedure. The measured intensities I(Q,c) are background corrected and scaled by concentration c followed by division with a structure factor *S´*(*Q,c*) to result in a form factor *P*(*Q,c*) = (*I*(*Q,c*)-*bgr*)/*c*/*S´*(*Q,c*). Form factors *P*(*Q,c*) for all concentrations yield the averaged form factor 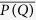. Structure factor parameters as size or charge, concentration and background contribution are iteratively optimized to minimize the squared relative deviation of the form factors from the average form factor χ^2^ = Σ_c_(P(Q,c)/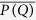
−1) to result in a self-consistent pair of averaged form factor 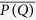
and structure factor parameters. Compared to the usual procedure of extrapolating the data to zero concentration to yield the form factor and subsequently the structure factor, this procedure results in lower errors for the form factor and the structure factor.

### Neutron Spin Echo Spectroscopy experimental setup

The NSE experiment was performed at J-NSE instrument, JCNS outstation at the MLZ Garching, Germany, at incident wavelengths of 8 Å, 10 Å and 12 Å accessing a dynamical range of 0.1 ≤ *t*_max_ ≤ 130 ns for several momentum transfers, *Q*, between 0.05 Å^−1^ and 0.2 Å^−1^. The entire detector area was summed up to reduce errors. The sample was loaded in a top loader quartz container of 40 × 30 × 4 mm and NSE spectra were taken at 25 °C. As a reference measurement, we used a Graphite powder sample loaded in an identical container and measured with the same instrument setup. Solvent buffer measurement was done under the same conditions with the dialysate for proper data reduction. Data analysis to yield the intermediate scattering function was performed with the ECHODET software package of the NSE instrument.

### Modelling and calculations

For atomic modelling we use MMTK from ref.^59^ to handle *pdb* structure files, access atomic coordinates and perform rotations and translations of subparts of the protein structure. SAXS scattering is calculated with a coarse graining on the amino acid level by assuming *Q* dependent scattering amplitudes for each amino acid including the contrast to the solvent buffer similar to ref.^57^. The NSE intermediate scattering function is calculated by coarse graining to 199 individual grains. Individual scattering amplitudes for each grain are calculated as the root of the grain form factors in sufficient accuracy compared to atomic resolution, similar to ref.^57^ for amino acids.

As a starting configuration, we use IgG-ALL.pdb of Padlan^37^ as shown in Fig. 1. Fragment bending flexibility in the linker is realized by two rotations around the axis through both Pro236-C_a_ atoms respectively Pro241-C_a_ of the heavy chains (*α*_236_, *α*_241_). These axes are approximately perpendicular to each other and located close to two disulfide bonds of IgG1 in the linker region. Additionally, the Fc fragment can move around the axis between both Pro247-C_a_ of the heavy chains to allow asymmetric deformation (*α*_247_). Stretching of the linker region is implemented by stretching all atom positions of both heavy chains with a stretching factor between Pro236-C_a_ and Pro246-C_a_ and each chain individually between Pro231-C_a_ atoms respectively Pro236-C_a_. Bending in the Fab fragments could be achieved by rotation around the axis defined by Ser228-C_a_ of the light and heavy chains. Specific positions for bending are arbitrarily chosen but represent positions where a good description of an overall flexibility can be expected. Coarse Graining is applied after application of bending and stretching.

### Brownian motion of the fragments in a harmonic potential

In the following, we consider Brownian motion in a harmonic potential around the equilibrium position 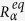, a problem described by the Ornstein-Uhlenbeck process^50^,^51^ and follow refs.^55^,^60^ in the derivation of the corresponding correlation function for coherent neutron scattering *F*_*coh*_(*Q,t*). Restricting the analysis to the internal coordinates of the protein, the translational and rotational degrees of freedom are separated. The rotational and translational degrees of freedom are described by rotational and translational diffusion, which can be treated within the decoupling approximation according to equation (5) and equation (6). The coherent intermediate scattering function *F*_*coh*_(*Q,t*) of atoms or subunits *α* with coherent scattering length b_α_ at positions R_α_ describing our internal dynamics can be written as


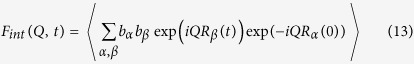


For larger subunits or coarse graining the scattering amplitude can be substituted by *Q* dependent scattering amplitude. With displacements 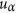 from the time independent equilibrium position 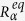 we can use 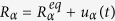 and 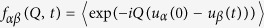 resulting in


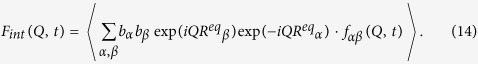


We describe the internal dynamics by a Langevin type equation as 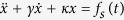 with position vector *x*, friction matrix *γ*, force constant matrix *κ* and random acceleration f_s_(t) in mass weighted coordinates and mass weighted friction and force constants^52^. In the case of vanishing friction (γ = 0), normal mode analysis with the eigenequation 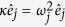 of eigenvalues 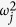 and eigenvectors 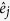 results in oscillating solutions describing vibrational motion. Here, we restrict ourselves to the highly over-damped case of high friction with a negligible acceleration term 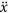. The high friction solution of Smoluchowski dynamics is solved by the eigenequation 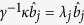 of eigenvalues *λ*_*j*_ and eigenvectors 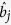 with characteristic exponentially decaying solutions. For details about the derivation of low and high friction limits see refs.^55^,^60^. According to the nature of the modes, the 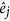 are called elastic or vibrational normal modes, while the 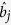 are called brownian normal modes indicating that the Brownian motion characterizes the modes and eigenvalues.

Using the normal modes we may decompose the last term *f*_*αβ*_(*Q,t*) in equation (14) in a constant term and a term describing the time dependence


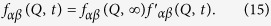


The constant term is related to the vibrational modes and only dependent on the harmonic potential as


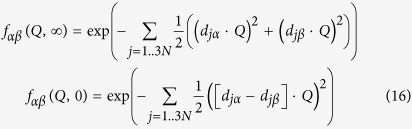


with vibrational displacement 
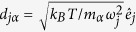
 of subunit *α* in normal mode *j*. *d*_*jα*_ is the displacement vector that corresponds to the width in a Gaussian distribution around equilibrium configuration 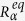 in the harmonic potential with force constant 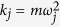 along normal mode *j*.

In the high friction limit the time dependent part within Smoluchowski dynamics is described by


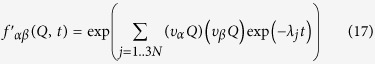


with displacements 
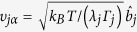
 of subunit *α* in Brownian normal mode *j* and friction 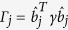. 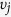 is the displacement vector within relaxation time 1/*λ*_*j*_. A force constant can be estimated by 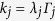. We assume the friction matrix *γ* to be diagonal and zero off diagonal terms^61^. For equal valued diagonal friction matrix, the vibrational and Brownian modes are equal and mode displacements can be used to calculate the displacement between the fragments. Friction with the solvent may be attributed to surface subunits, but can also be equally distributed for rigid domains for simplicity. For independent relaxing modes the mean square displacement is 
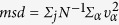
.

The coherent intermediate scattering function describing the internal dynamics is finally


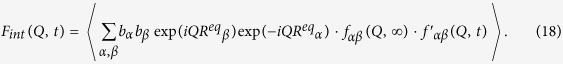


The contribution of internal dynamics to the NSE signal can be calculated accordingly by


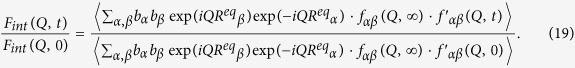


The close similarity of numerator and denominator let us perceive that the dominating term describing the time evolution is of the form exp(*−~*exp(*−t*)). In fact, for the case of a single independent particle in a harmonic trap, the dynamics can be described by the much simpler function shown in equ. (12).

## Additional Information

**How to cite this article**: Stingaciu, L. R. *et al.* Fast antibody fragment motion: flexible linkers act as entropic spring. *Sci. Rep.* **6**, 22148; doi: 10.1038/srep22148 (2016).

## Supplementary Material

Supplementary Information

## Figures and Tables

**Table 1:** Resulting fit parameters according to different models.

model	dof 9	dof 6	msd
relaxation time [ps]	6300 ± 1100	6600 ± 1100	7500 ± 1700
rmsd per mode [nm]	0.39 ± 0.04	0.48 ± 0.04	0.44 ± 0.03
friction per atom [g/ps/mol]	5.7 ± 0.4	3.9 ± 0.3	
friction per fragment (Fc/Fab) [g/ps/mol]	41100 ± 2900/37100 ± 2600	28300 ± 2200/25500 ± 2000	
force constant of fragment (Fc/Fab [g/ps^2^/mol]	6.5 ± 1.2/5.9 ± 1.1	4.2 ± 0.8/3.8 ± 0.7	12.8
force constant of fragment (Fc/Fab)[pN/nm]	10.9 ± 2.0/9.8 ± 1.8	7.1 ± 1.3/6.4 ± 1.2	21.2
H_R_	0.82 ± 0.05	0.77 ± 0.06	0.77 ± 0.07

*dof* 9 is fitted with 9 degrees of freedom, *dof* 6 is fitted with 6 degrees of freedom due to restricted motion along the linker, *msd* is the simplified model according to equation (12). Conversion to pN/nm = 1.66 g/ps^2^/mol is given for easier comparison with other work
