# Supplementary material for: Fast antibody fragment motion: flexible linkers act as entropic spring
Source: Sci Rep. 2016 Mar 29;6:22148. doi: 10.1038/srep22148 (PMC4810366; doi:10.1038/srep22148)
Supplement: Supplementary Information [file srep22148-s1.pdf]

Fast antibody fragment motion: flexible linkers act as entropic spring

Laura R. Stingaciu, Oxana Ivanova, Michael Ohl, Ralf Biehl, Dieter Richter

Supporting information

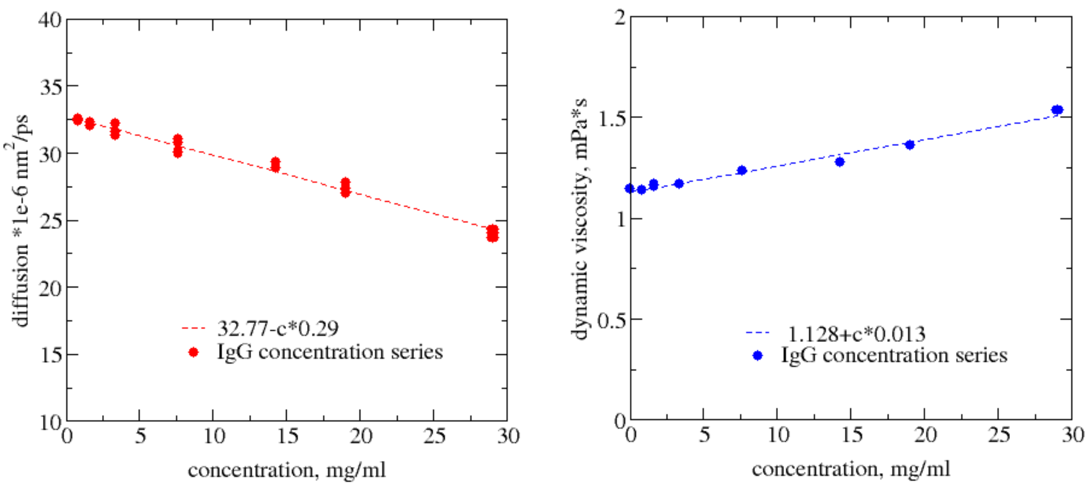

Figure S1. (a) Translational diffusion of IgG concentration series measured by DLS. Red dashed line is extrapolation to zero concentration. D_{T0} = 3.27e-7 cm^2/s. (b) Dynamic viscosities of IgG concentration series. Blue dashed line is extrapolation to zero concentration. η_0 fitted value of 1.128 mPa·s is comparable with η_0 experimentally measured for solvent solution at 25°C (zero IgG concentration) = 1.147 mPa·s.

Dynamic Light Scattering (DLS) experimental diffusion coefficients of IgG concentration series between 0.8 mg/ml and 29 mg/ml are show in Fig. S1a. By extrapolating to zero concentration, we obtained D_{T0} = 3.27e-7 cm^2/s.

The average translational hydrodynamic function H_T can be calculated from the intrinsic viscosity $[\eta]$ by: $H_T = 1 - c[\eta]$. For this purpose, we measured the dynamic viscosities η for IgG concentration series between 0.8 mg/ml and 29 mg/ml. These are shown in Fig. S1b. The intrinsic viscosity of the highest IgG concentration 29 mg/ml was determined to value $[\eta] = (\eta - \eta_0) / \eta_0 c$ = 0.0116 where η_0 is the buffer measured viscosity (0.01147 Poise). For this value of the intrinsic viscosity, we calculate the average translational hydrodynamic function H_T = 0.66.
